# Anomalous Origin of the Left Coronary Artery From the Right Coronary Cusp: A Case Report

**DOI:** 10.7759/cureus.35711

**Published:** 2023-03-03

**Authors:** Jurgen Shtembari, Dhan B Shrestha, Prakash R Oli, Anish Munagala, Elda Mullaj, Kerolus Shehata, Daniela Kovacs, Sandeep Khosla

**Affiliations:** 1 Department of Internal Medicine, Mount Sinai Hospital, Chicago, USA; 2 Department of Medicine, Mount Sinai Hospital, Chicago, USA; 3 Department of Medicine, Province Hospital, Surkhet, NPL; 4 Department of Cardiology, Mount Sinai Hospital, Chicago, USA

**Keywords:** coronary angiogram, anomalous coronary artery, lca, rca, stemi

## Abstract

Anomalous origin of the left main coronary trunk from the right coronary sinus is a rare condition and is associated with a significantly increased risk of cardiac events, including sudden cardiac death, and it may pose difficulties in their management using revascularization strategies. We present a case of a 68-year-old man with worsening chest pain. Initial evaluation revealed ST elevation of the inferior wall leads and elevated troponins. He was diagnosed with ST-elevation myocardial infarction (STEMI) and sent for emergency cardiac catheterization. Coronary angiography showed 50% stenosis of the mid-right coronary artery (RCA) that extended as a total occlusion to the distal RCA and an unexpected anomalous origin of the left main coronary artery (LMCA). Our patient's LMCA originated from the right cusp sharing a single ostium with the RCA. Multiple attempts of revascularization with percutaneous coronary intervention (PCI), using multiple wires, catheters, and different-sized balloons, were unsuccessful due to complex anatomy. Our patient was managed with medical therapy and discharged home with close cardiology follow-up.

## Introduction

The incidence of the left main trunk originating from the right sinus of Valsalva is a rare condition with an incidence of 0.017% [1]. Among different coronary artery anomalies, an anomalous coronary artery from the opposite sinus of Valsalva (ACAOS) has a higher risk of angina pectoris, myocardial infarction, cardiac failure, dysrhythmia, and sudden cardiac death (SCD) [1-3]. The risk of cardiac death due to ACAOS is higher in patients with a left coronary artery (LCA) originating from the right coronary sinus [2]. We present a case of the left main coronary trunk arising from the right coronary cusp in a 68-year-old man who presented with acute ST-elevation myocardial infarction (STEMI).

## Case presentation

**Figure 1:**
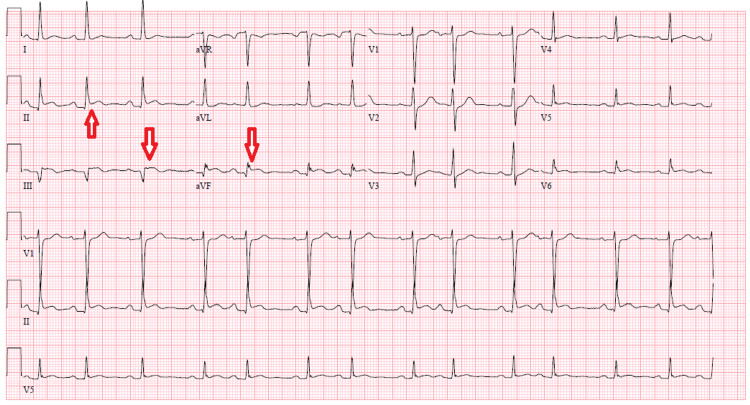
A 12-lead electrocardiograph showing ST-segment elevation in leads II, III, and aVF (red arrows).

A 68-year-old man with diabetes and hypertension presented to the Emergency Department (ED) with left-sided typical chest pain that he has been experiencing for the past three weeks. He reported worsening pain in the last two days after smoking cocaine. His pain was not relieved by ibuprofen, and he also reported associated shortness of breath, sweating, and nausea without vomiting. His electrocardiogram (ECG) showed ST elevation in inferior leads (Figure 1). Initial workup revealed elevated high sensitivity troponin I and brain natriuretic peptide and a normal chest X-ray. He received a loading dose of aspirin 325 mg and ticagrelor 180 mg for STEMI and was taken for emergency cardiac catheterization.

**Figure 2:**
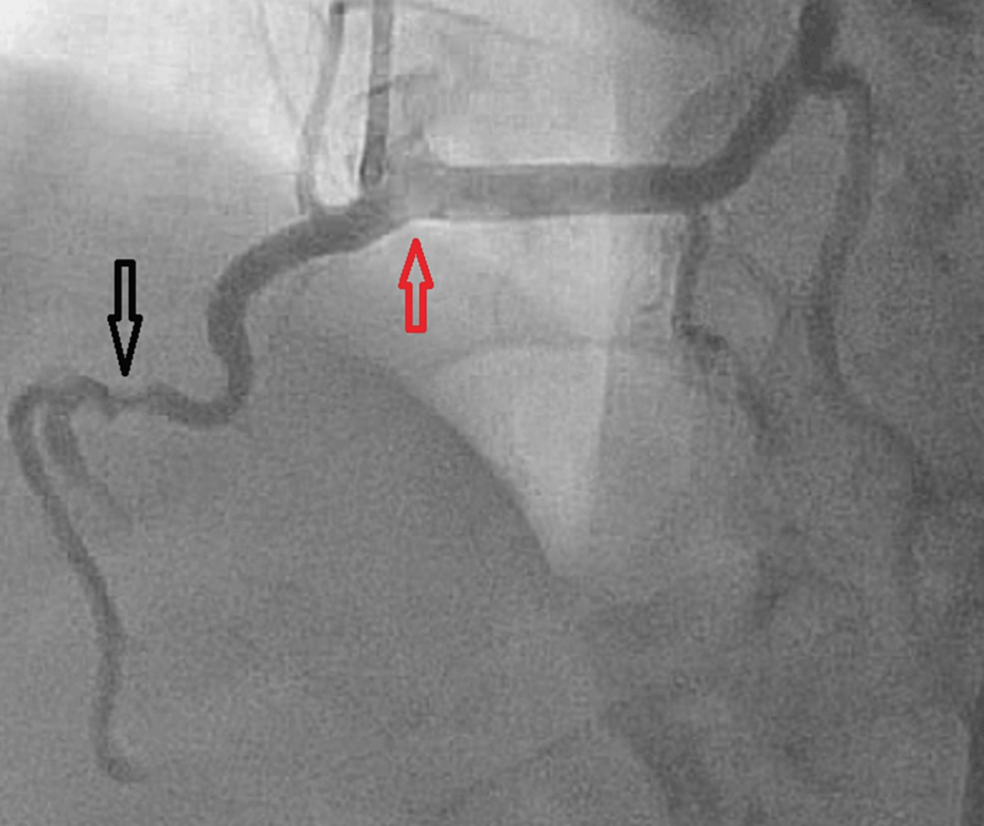
Coronary angiogram showing the left main coronary artery originating from the right coronary cusp along with RCA (red arrow) and stenosis of the mid-RCA (black arrow). RCA, right coronary artery

**Figure 3:**
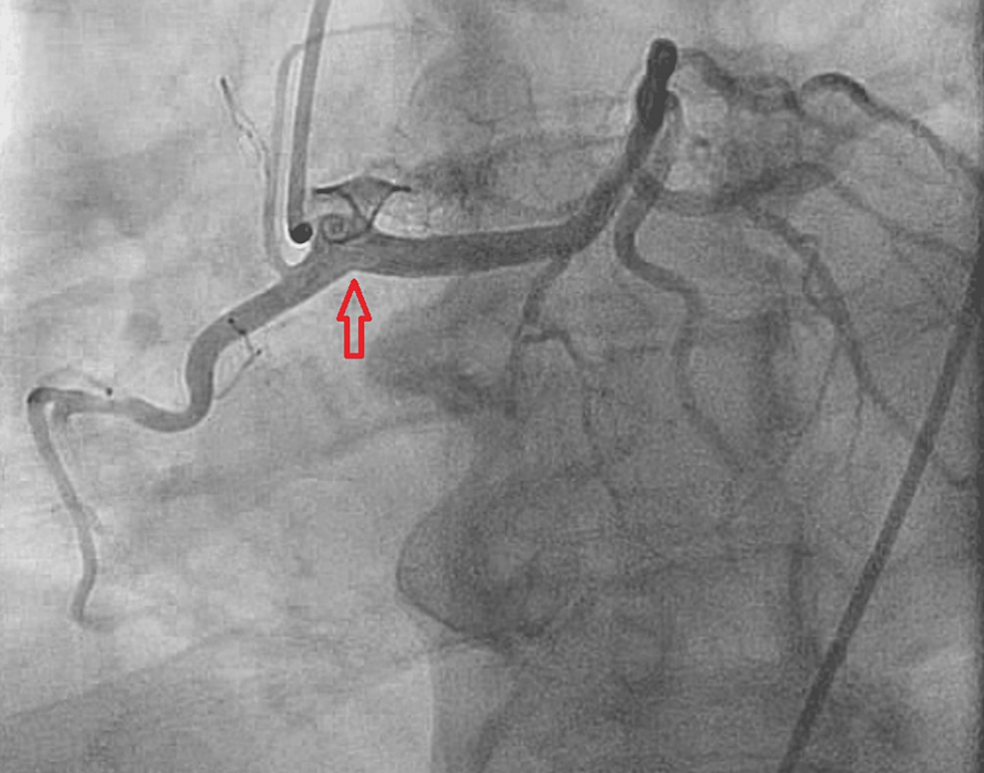
Coronary angiogram showing the left main coronary artery originating from the right coronary cusp along with the RCA (red arrow). RCA, right coronary artery

Emergency coronary angiography revealed a 50% stenosis of the mid-right coronary artery (RCA), with 100% acute on chronic stenosis of the distal RCA and a calcified, anomalous origin of the left main coronary artery coming from the right cusp and sharing a single ostium with the RCA (Figures 2-3).

Due to complicated anatomy, the intervention was aborted after multiple unsuccessful attempts to place a balloon across the lesion. The patient had residual 100% stenosis with thrombolysis in myocardial infarction (TIMI) grade 0 flow, and the patient opted to be treated medically. Echocardiography showed a normal-sized left ventricle (LV) cavity with normal LV systolic function with ejection fraction (EF) of 60% to 65%, and hypokinesis of the inferior myocardium. This complex lesion was discussed with the Heart Team. Following interdisciplinary recommendations, our patient was optimized with guideline-directed medical therapy for STEMI and was counseled on the cessation of cocaine and tobacco. He was referred to cardiac rehabilitation and discharged with close cardiology and cardiothoracic surgery follow-up. He underwent a coronary artery bypass graft (CABG) with a triple vessel bypass in another facility. He was briefly readmitted to our center for deconditioning following CABG and cardiac rehabilitation, and he preferred to continue physical therapy at a skilled nursing facility.

## Discussion

The anomalous origin of coronary arteries is an uncommon finding. In a study by Yamanaka and Hobbs, among 126,595 patients who had coronary angiography, the incidence of congenital coronary artery anomalies was 1.3%, 87% of the cases had an anomalous origin or distribution, and 2.3% of all anomalies included a left main trunk originating from the right sinus of Valsalva [1]. Similarly, in a study among continuous series of coronary angiograms by Angelini in 2007, the incidence of ACAOS was 1.07% - 0.92% incidence of anomalous origin of the RCA from the left sinus and 0.15% incidence of anomalous origination of the LCA from the right sinus [4]. The anomalous LCA origin can be categorized into five subtypes based on its location in relation to the aorta and the pulmonary trunk. Among the five subtypes, the left main trunk passing between the aorta and the pulmonary trunk increases the risk of serious complications such as angina pectoris, myocardial infarction, syncope, and ventricular tachycardia, despite the absence of coronary atherosclerosis [1].

Our patient had multiple cardiac risk factors, which accelerated the pathophysiology of coronary atherosclerosis and acute coronary syndrome, resulting in total occlusion of the RCA leading to STEMI in the background of the anomalous left main trunk origin, which is a coincidental finding.

In 2018, the American Heart Association (AHA)/American College of Cardiology (ACC) recommended coronary angiography using catheterization or imaging to evaluate anomalous coronary arteries. They also recommend performing the anatomic and physiological evaluation of patients with the anomalous aortic origin of the left coronary from the right sinus or vice versa [5]. In our case, the emergency coronary angiography depicted the anomalous origin of the left main coronary artery from the right coronary cusp along with the right coronary artery and stenosis of the right coronary artery.

With the paucity of conditions and their associated knowledge, there are dilemmas justifying which treatment option is best for a patient. There are three possible treatment modalities in patients with ACAOS: medical treatment/observation, coronary angioplasty with stent deployment, and surgical repair. Medical management using beta blockers is as effective as limited provocative actions [4,5].

Most anomalous coronary artery origins are benign and require no treatment, while five types may warrant some form of intervention to prevent SCD and other complications. These subtypes are (1) the asymptomatic or symptomatic anomalous aortic origin of the coronaries; (2) anomalous aortic origin of the LCA; (3) anomalous aortic origin of the right coronary artery; (4) large coronary fistula anomalous left coronary from the pulmonary artery; and (5) anomalous right coronary from the pulmonary artery [6]. Our patient's anomaly was a second subtype anomaly that requires surgical intervention as per the AHA/ACC guidelines. The morphology of his anatomy exposes our patient to an increased risk for future cardiac events. It is a class I indication to perform a surgical intervention in the subgroup of patients with LCA originating from the right sinus and ischemic symptoms or stress-induced ischemia and a class IIa indication for asymptomatic patients [5]. Due to the failure of percutaneous intervention and our patient opting for medical management, he was discharged with close outpatient cardiothoracic surgery and cardiology appointments for a possible further intervention soon. The patient was found to have CABG in another facility.

Oliveira et al. employed a similar approach to managing medical therapy for their patient, and there was no recurrence of angina and major cardiovascular or cerebrovascular events [7]. In a case report by Gac et al., they opted for medical therapy amid interventional and surgical treatment considering the nature of the pathophysiology and comorbidities of their patient despite considering CABG as a potential option [8]. Percutaneous coronary intervention (PCI) and surgical intervention as effective treatment options are well illustrated by Lee and Park [9], Khalighi et al. [2], Narayanan et al. [3], and Baik et al. [10].

## Conclusions

The left main trunk arising from the right coronary cusp is a rare congenital anomaly associated with an increased risk of cardiac events and sudden deaths. In a patient with coronary artery disease, these anomalies may pose difficulties in their management using revascularization strategies.
